# A Meta-Analysis of Adenosine A2A Receptor Antagonists on Levodopa-Induced Dyskinesia *In Vivo*

**DOI:** 10.3389/fneur.2017.00702

**Published:** 2017-12-22

**Authors:** Wen-Wen Wang, Man-Man Zhang, Xing-Ru Zhang, Zeng-Rui Zhang, Jie Chen, Liang Feng, Cheng-Long Xie

**Affiliations:** ^1^The Center of Traditional Chinese Medicine, The Second Affiliated Hospital, Yuying Children’s Hospital of Wenzhou Medical University, Wenzhou, China; ^2^Department of Neurology, The First Affiliated Hospital of Wenzhou Medical University, Wenzhou, China

**Keywords:** adenosine, A2A receptor antagonists, levodopa-induced dyskinesia, Parkinson’s disease, meta-analysis

## Abstract

**Background:**

Long-term use of levodopa (l-dopa) is inevitably complicated with highly disabling fluctuations and drug-induced dyskinesias, which pose major challenges to the existing drug therapy of Parkinson’s disease.

**Methods:**

In this study, we conducted a systematic review and meta-analysis to assess the efficacy of A2A receptor antagonists on reducing l-dopa-induced dyskinesias (LID).

**Results:**

Nine studies with a total of 152 animals were included in this meta-analysis. Total abnormal involuntary movements (AIM) score, locomotor activity, and motor disability were reported as outcome measures in 5, 5, and 3 studies, respectively. Combined standardized mean difference (SMD) estimates were calculated using a random-effects model. We pooled the whole data and found that, when compared to l-dopa alone, A2A receptor antagonists plus l-dopa treatment showed no effect on locomotor activity (SMD −0.00, 95% confidence interval (CI): −2.52 to 2.52, *p* = 1.0), superiority in improvement of motor disability (SMD −5.06, 95% CI: −9.25 to −0.87, *p* = 0.02) and more effective in control of AIM (SMD −1.82, 95% CI: −3.38 to −0.25, *p* = 0.02).

**Conclusion:**

To sum up, these results demonstrated that A2A receptor antagonists appear to have efficacy in animal models of LID. However, large randomized clinical trials testing the effects of A2A receptor antagonists in LID patients are always warranted.

## Introduction

Parkinson’s disease (PD) is defined by a set of motor signs and symptoms that are caused by selective degeneration of the dopamine (DA) neurons, which originate in the substantia nigra pars compacta and project into the striatum (1). The most efficacious treatment for PD is the DA precursor levodopa (l-dopa), which not only improves all typical parkinsonian motor symptoms but also increases patients’ life expectancy (2). However, the long-term use of l-dopa produces motor complications which include highly disabling fluctuations and l-dopa-induced dyskinesias (LID), representing the major challenges to the existing drug therapy of PD (3, 4). The management of LID includes switching to a controlled-release l-dopa or adding a COMT inhibitor/monoamineoxidase B inhibitor/a longer acting DA agonist (5). However, once LID is established, the increase in dopaminergic load resulted from these strategies can only lead to an aggravation of the condition, not only in the severity but also the duration (6). Moreover, recent researches have revealed that a wide series of non-dopaminergic neurotransmitter systems (glutamatergic, serotoninergic, adrenergic, and cholinergic, etc.) are involved in pre- and postsynaptic changes and thereby contributing to the pathophysiology of LID (5). Based on such therapies, many drugs are available in the market to treat disabling LID, but potential side effects have limited their clinical use (7).

The general pathophysiological interpretation and various hypotheses have been discussed for the genesis and development of LID. However, the exact mechanisms and the molecular targets are still poorly understood (8). Adenosine is believed to play a neuroprotective role in central neurodegenerative disorders and its actions are mediated by different receptors such as A1, A2A, A2B, and A3 (9). It has been suggested that A2A receptors are highly localized to the basal ganglia nuclei of the indirect output pathway, where they are co-localized with DA D2 receptors, and may be capable of influencing motor activity by acting at different levels (10). It is hypothesized that the effects of adenosine A2A receptor antagonists on motor function is attributed to their inhibitory function on neurons of the indirect pathway that expressing both A2A and D2 receptors (10). Moreover, inactivation A2A receptors on striatopallidal neurons of the indirect pathway can produce a parallel behavioral activation by means of mimicking the motor stimulant actions of co-localized D2 receptors on these neurons (11). Preclinical behavioral investigations reported that pharmacological antagonism or genetic knockdown of A2A receptors may be of interest to the management of LID (12). Furthermore, results from clinical studies had also shown that A2A receptor antagonists’ significantly reduced off-time as an adjunctive therapy to l-dopa in advanced PD patients with motor fluctuations, also suggesting a possible reduced risk of l-dopa-induced motor complications (13). However, to date, few clinical trials have tested the benefits of A2A antagonists in LID patients. Limited clinical data demonstrate the possibility that in PD patients with established dyskinesia, we might be able to maintain the anti-parkinsonian response and reduce dyskinesia by adding an A2A antagonist and lowering the l-dopa dose, though this remains to be proven (14).

Systematic reviews and meta-analyses of preclinical animal data could facilitate the planning of further investigations and improve the likelihood of success of future clinical trials, also identify where there is a need for further experiment research, preclude unnecessary study replication, and contribute to both reduction and refinement in animal experimentation (15). Therefore, we report a systematic review and meta-analysis of adenosine A2A receptor antagonists in experimental models of PD with LID.

## Materials and Methods

The whole process and methods of this meta-analysis were performed according to our previous published paper (16) and based on the modified Preferred Reporting Items for Systematic Reviews and Meta-Analyses (PRISMA) Statement.

### Search Strategy

We electronically searched three databases (PubMed, Google scholar, and EMBASE database) up to January 2016 for all publications written in English. All searches were limited to studies on animals. Reference lists from the included literature were scrutinized to identify further relevant publications. The search strategy as follows:

1. adenosine
2. adenocard
3. adenoscan
4. or/1–3
5. dyskinesia
6. abnormal
7. involuntary movements
8. hemiballismus
9. or/5–8
10. 4 and 9

### Inclusion and Exclusion Criteria

Inclusion criteria:

(1) Test the effects of adenosine A2A receptor antagonists on LID in animal models of PD. Adenosine A2A receptor antagonists are defined as a drug with reported agonism on at least one class of A2A receptor irrespective of actions at other receptor classes.
(2) Use abnormal involuntary movements (AIM) or neurobehavioral score as the outcome measure comparing LID animals receiving combined treatment (both adenosine A2A receptor antagonists and l-dopa) and l-dopa/benserazide or l-dopa/carbidopa alone. AIM ratings were performed as we previously described (17). Briefly, 0 = absent, 1 = present less than 50% of the observation period, 2 = present more than 50% of the observation time, 3 = present all the times but suppressible by external stimuli, and 4 = present all the times and not interfering by external stimuli.
(3) Original data being independent of other studies.

Pre-specified exclusion criteria were: (1) case reports, abstracts, comments, reviews, editorials, and clinical trials; (2) dyskinesia was not developed by long-term l-dopa; (3) not testing the efficacy of adenosine receptor antagonists on LID; and (4) AIM or neurobehavioral score was not the outcome measure.

### Data Extraction

From included studies, data listed out as follows were extracted using a comprehensive approach: (1) the first author’s name and publication year, experimental models [1-methyl-4-phenyl-1,2,3,6-tetrahydropyridine (MPTP) models or 6-Hydroxydopamine (6-OHDA) models, etc.]; (2) individual data for each animal including number, species, sex, weight, and anesthetic used; (3) information on treatment including route of administration and dosage; and (4) outcome measures and time of assessment of outcomes. If outcomes were presented at different time points, we extracted data from the last one reported. If the data for meta-analysis were missing or only expressed graphically, we tried to contact the authors for further information, or calculate by ourselves using digital ruler software, or excluded the study which we could not get enough information. For each comparison, we extracted data of mean value and standard deviation from treatment and control group, respectively, of each study.

### Quality Assessment

Study quality was evaluated based on a six-item modified scale (18): peer-reviewed publication, random allocation to groups, blinded assessment of outcome, sample size calculation, compliance with animal welfare regulations, and a statement of a potential conflict of interest. For the calculation of an aggregate quality score, each item of the six-item modified scale was attributed one point. Two authors (Cheng-Long Xie and Jie Chen) independently extracted data and assessed study quality. Disagreements were solved after discussion on the details of the studies.

### Statistical Analysis

We utilized a random-effects model because it took into account the fact that the true treatment effects had likely varied among the included trials. We conceived the main outcome measures as continuous data, and were given an estimate of the combined overall effect sizes using standardized mean difference (SMD) and its standard error, with 95% confidence interval (CI). To pool different scales, we used the SMD as the summary statistic in our meta-analysis as it reveals the effect size of the treatment relative to the variability observed in the same study (19). For the assessment of heterogeneity, the *I*^2^ statistic was used. To assess the stability of the results, a sensitivity analysis was performed by removing each individual study in turn from the total and reanalyzing the remainder. Meanwhile, we performed a stratified meta-analysis with experiments grouped according to different kinds of adenosine A2A receptor antagonists. All the analyses were done with Revman software 5.0. Probability value *p* < 0.05 was considered significant.

## Results

### Results of the Search

**Figure 1:**
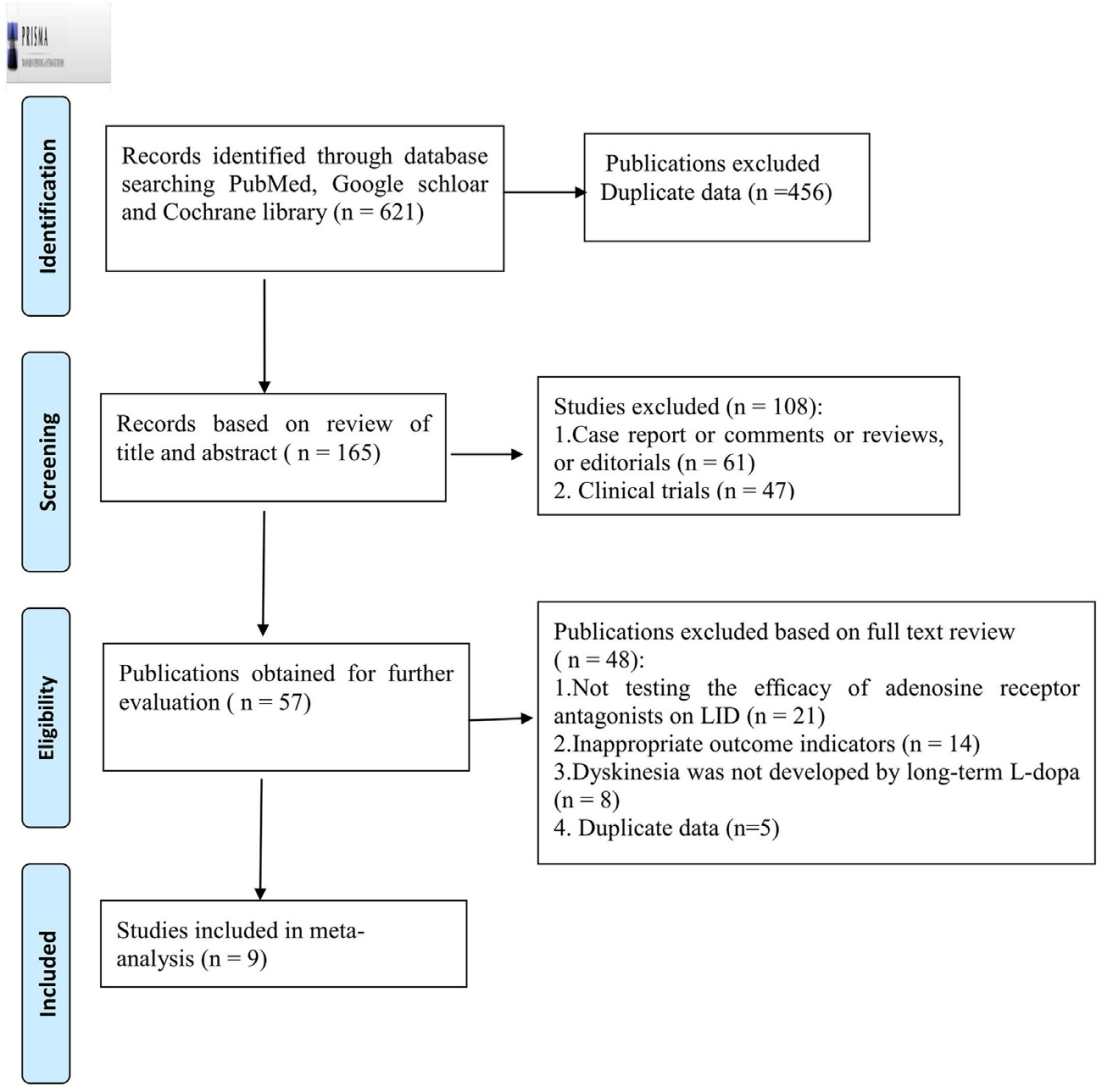
PRISMA 2009 Flow Diagram of trial identification, inclusion, and exclusion. LID, levodopa-induced dyskinesia.

From the electronic search, 621 publications were initially identified, from which we excluded 456 due to repetition. After screening the titles and abstracts, 108 were further excluded because they failed to meet the inclusion criteria. By reading the full text of the remaining 57 articles, 40 studies were excluded as a result of not testing the efficacy of adenosine A2A receptor antagonists on LID (*n* = 21), inappropriate outcome indicators (*n* = 14), and the problem of duplicate data (*n* = 7). Another eight studies were excluded as the dyskinesia presented was not developed by long-term l-dopa. Ultimately, nine eligible studies were identified (12, 20–27) (Figure 1).

### Study Characteristics

These studies involved 152 animals (A2A receptor antagonists 83, control 69) from three species: Mice (*n* = 53), Sprague-Dawley rats (*n* = 67), and Marmosets (*n* = 32), respectively. The studies varied in size, ranging from 8 to 36 animals. Three out of nine (3/9, 33.3%) studies were MPTP models, while the remaining utilized the 6-OHDA models (6/9, 66.7%). The studies were published between 2000 and 2014. These studies included animals of both sexes (*n* = 4), ^22,23,24,25^ male only (*n* = 3), ^18,19,23^ female only (*n* = 1), ^21^ and gender not reported (*n* = 1) (12). To induce LID in PD model, animals were treated, once parkinsonism was stable, with twice-daily administration of l-dopa (2–25 mg/kg, i.p.) plus benserazide/carbidopa (2.5–15 mg/kg, i.p.) for several weeks, ranging from 16 days to 8 weeks. KW-6002 as the A2A receptor antagonist was reported in four studies (12, 20, 21, 27), ST1535 (22, 23) in two, Caffeine (24, 25) in two, and SCH 412348 (26) in one study. Meanwhile, total AIM score, locomotor activity, and motor disability were reported as a outcome measure in 5, 5, and 3 studies, respectively. The basic characteristics of the nine selected studies are summarized in Table 1.

**Table 1:** Basic characteristics of the included studies.

Reference	Species, weight, anesthetic	Model (Parkinson’s disease/l-dopa-induced dyskinesias)	Intervention	Outcome index	*p*-Value
**6-0HDA models**					
Lundblad et al. (21)	SD rats (female, 9/10), 225 g, NR	Unilateral 6-OHDA (8 μg/3 μl) lesioned; l-dopa (4 mg/kg)/carbidopa (12 mg/kg) for 16 days	KW-6002 (3 and 10 ml/kg, p.o.) for 16 days	1. Rotarod performance 2. Total AIM	1. *p* > 0.05 2. *p* < 0.05
Danqing (12)	Mice (NR,10/7), NR, avertin	Unilateral 6-OHDA (8 μg/4 μl) lesioned; l-DOPA (2 mg/kg)/benserazide (2 mg/kg) for 21 days	KW-6002 (0.03 mg/kg and 0.3 mg/kg, i.p.) for 21 days	1. Locomotor Activity 2. Total AIM	1. *p* > 0.05 2. *p* < 0.05
Elisabetta (23)	SD rats (male, 8/8), 275–300 g, chloral hydrate	Unilateral 6-OHDA (8 μg/4 μl) lesioned; l-DOPA (3 mg/kg)/benserazide (6 mg/kg) for 18 days	ST1535 (20 mg/kg, p.o.) for 18 days	1. Locomotor Activity 2. limb and axial AIM	1. *p* > 0.05 2. *p* < 0.05
Yi-xian (24)	SD rats (male, 10/10), 180–220 g, chloral hydrate	Unilateral 6-OHDA (8 μg/4 μl) lesioned; l-dopa (25 mg/kg)/benserazide (6.25 mg/kg) for 21 days	Caffeine (2.5 mg/kg, i.p.) for 21 days	1. Rotational response 2. Total AIM, limb AIM 3. Orolingual AIM	1. *p* > 0.05 2. *p* < 0.05 3. *p* < 0.05
Danqing (25)	Mice (both sex, 24/12), NR, avertin	Unilateral 6-OHDA (10 μg/4 μl) lesioned; l-dopa (2 mg/kg)/benserazide (2 mg/kg) for 21 days	Caffeine (3 and 15 mg/kg, i.p.) for 21 days	1. Total AIM	1. *p* < 0.05
Jones et al. (26)	SD rats (male, 6/6), 350–400 g, NR	Unilateral 6-OHDA (8 μg/4 μl) lesioned; l-dopa (6 mg/kg)/benserazide (15 mg/kg) for 22 days	SCH 412348 (3 mg/kg, i.p.) for 22 days	1. Catalepsy 2. Total AIM	1. *p* > 0.05 2. *p* > 0.05
**MPTP models**					
Tomoyuki (20)	Marmosets (both sex, 4/4), 285–420 g, NR	MPTP 2.0 mg/kg sc for 5 days; l-dopa (12.5 mg/kg)/carbidopa (12.5 mg/kg) for 21 days	KW-6002 (10 ml/kg, p.o.) for 21 days	1. Locomotor Activity 2. Motor disability	1. *p* > 0.05 2. *p* < 0.05
Sarah (22)	Marmosets (both sex, 4/4), 265–437 g, NR	MPTP 2.0 mg/kg sc for 5 days; l-dopa (2.5 mg/kg)/carbidopa (12.5 mg/kg) for 8 weeks	ST1535 (20 mg/kg, p.o.) for 8 weeks	1. Locomotor Activity 2. Motor disability	1. *p* > 0.05 2. *p* < 0.05
Shin-ichi (27)	Marmosets (both sex, 8/8), 285–420 g, NR	MPTP 2.0 mg/kg sc for 5 days; l-dopa (10 mg/kg)/carbidopa (2.5 mg/kg) for 28 days	KW-6002 (10 mg/kg, p.o.) for 28 days	1. Locomotor Activity 2. Motor disability	1. *p* > 0.05 2. *p* < 0.05

*AIM, abnormal involuntary movements; SD rats, Sprague-Dawley rats; 6-OHDA, 6-hydroxydopamine; NR, no report; MPTP, 1-methyl-4-phenyl-1, 2, 3, 6-tetrahydropyridine; MFB, medial forebrain bundle; l-dopa, levodopa*.

### Risk of Bias

Of whom, one study got 6 points (1/9, 11.1%), four studies got 5 (4/9, 44.4%), three studies got 4 (3/9, 33.3%), and one study got 3 (1/9, 11.1%). Only one study described a sample size calculation. Random allocation to a treatment group and blinded assessment of outcome were described in seven studies. Eight studies mentioned the statement of potential conflict of interests. All studies reported compliance with animal welfare regulations (Table 2). Generally, all of the included studies were deemed to have a low risk of bias.

**Table 2:** Risk of bias of included studies.

Reference	Tomoyuki (20)	Lundblad (21)	Danqing (12)	Sarah (22)	Elisabetta (23)	Yi-xian (24)	Danqing (25)	Jones et al. (26)	Shin-ichi (27)
A	Y	Y	Y	Y	Y	Y	Y	Y	Y
B	Y	Y	Y	N	Y	Y	N	Y	Y
C	N	Y	Y	Y	Y	N	Y	Y	Y
D	N	N	Y	N	N	N	N	N	N
E	Y	Y	Y	Y	Y	Y	Y	Y	Y
F	Y	Y	Y	N	Y	Y	Y	Y	Y
Total	4	5	6	3	5	4	4	5	5

*Studies fulfilling the criteria of: A: peer-reviewed publication; B: random allocation to group; C: blinded assessment of outcome; D: a sample size calculation; E: compliance with animal welfare regulations; and F: a statement of a potential conflict of interest. Y, yes; N, no*.

### Meta-Analyses

**Figure 2:**
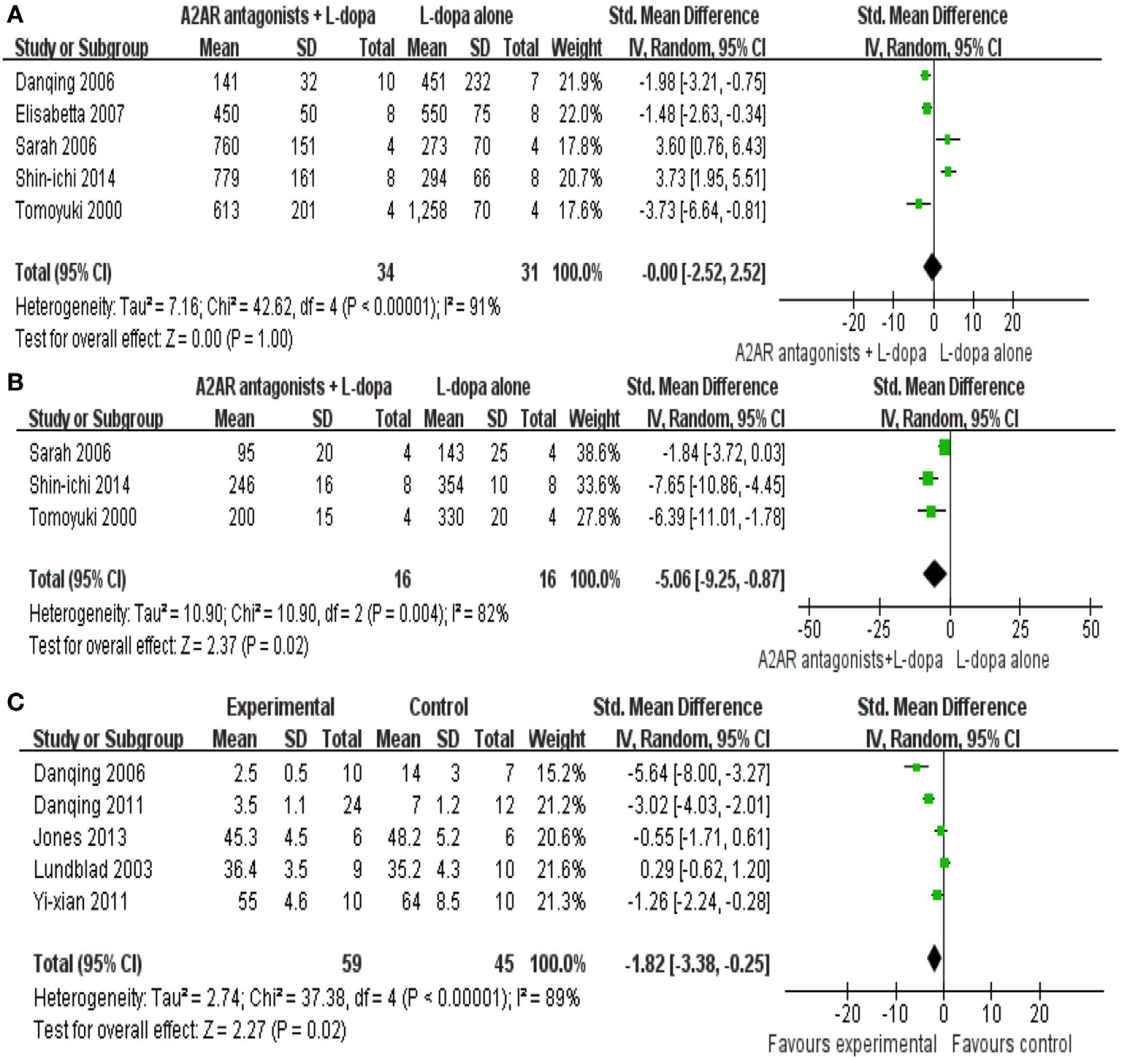
Total effect size of A2A receptor antagonists on locomotor activity score **(A)** reduction including 65 animals in five comparisons, motor disability score outcome **(B)** improvement (32 animals in three comparisons) and total abnormal involuntary movements (AIM) score **(C)** comprising 104 animals in five comparisons.

All the data for meta-analysis were expressed graphically. We used digital ruler software to calculate the mean and standard error. In the present paper, five studies reported the locomotor activity as the outcome measure with 67 animals were included in the final meta-analyses. We pooled the whole data and found no significant difference between A2A receptor antagonists plus l-dopa treatment and l-dopa alone (SMD −0.00, 95% CI: −2.52 to 2.52, *p* = 1.0, Figure 2A). Meanwhile, there was obvious heterogeneity for the analysis of locomotor activity between studies (Tau^2^ = 7.16, Chi^2^ = 42.62, *p* < 0.00001, *I*^2^ = 87%, Figure 2A). After sequentially excluding each study, the results of heterogeneity and locomotor activity were consistent (*I*^2^ range from 80 to 92%; *p* = 0.56 to 1.53 > 0.05, respectively). Consequently, we should interpret the pool result prudently.

Three studies reported mild significant effects of A2A receptor antagonists plus l-dopa treatment for improving motor disability compared with l-dopa alone (SMD −5.06, 95% CI: −9.25 to −0.87, *p* = 0.02, Figure 2B). Nevertheless, there was severe heterogeneity for the analysis of motor disability between studies (Tau^2^ = 10.90, Chi^2^ = 10.90, *p* = 0.004, *I*^2^ = 82%, Figure 2B). Removal of the outlier studies (22) led to more homogeneous results (Tau^2^ = 0.00, Chi^2^ = 0.19, *p* = 0.66, *I*^2^ = 0%), as well as increased the effect size by −2.18, yielding a still significant pooled SMD of −7.24.

Total AIM was reported by five trials, the result showed a significant difference in the reduction of the score between the A2A receptor antagonists plus l-dopa treatment and l-dopa alone (SMD −1.82, 95% CI: −3.38 to −0.25, *p* = 0.02, Figure 2C). Nevertheless, inspection of the data showed that the heterogeneity for the analysis of total AIM between studies was high (Tau^2^ = 2.74, Chi^2^ = 37.38, *p* < 0.00001, *I*^2^ = 89%). After sequentially excluding each study, the results of heterogeneity were consistent with the previous result. The remaining four studies (20, 22, 23, 27) failed to enter the pool analysis due to the lack of a Total AIM, one using limb and axial AIM to reflect LID, two using locomotor activity as the outcome measures, and the last one not reporting the criteria to assess the dyskinesia. Funnel plots were not applied to test the publication bias in this paper as the numbers of included studies were small.

### Subgroup Analysis

**Figure 3:**
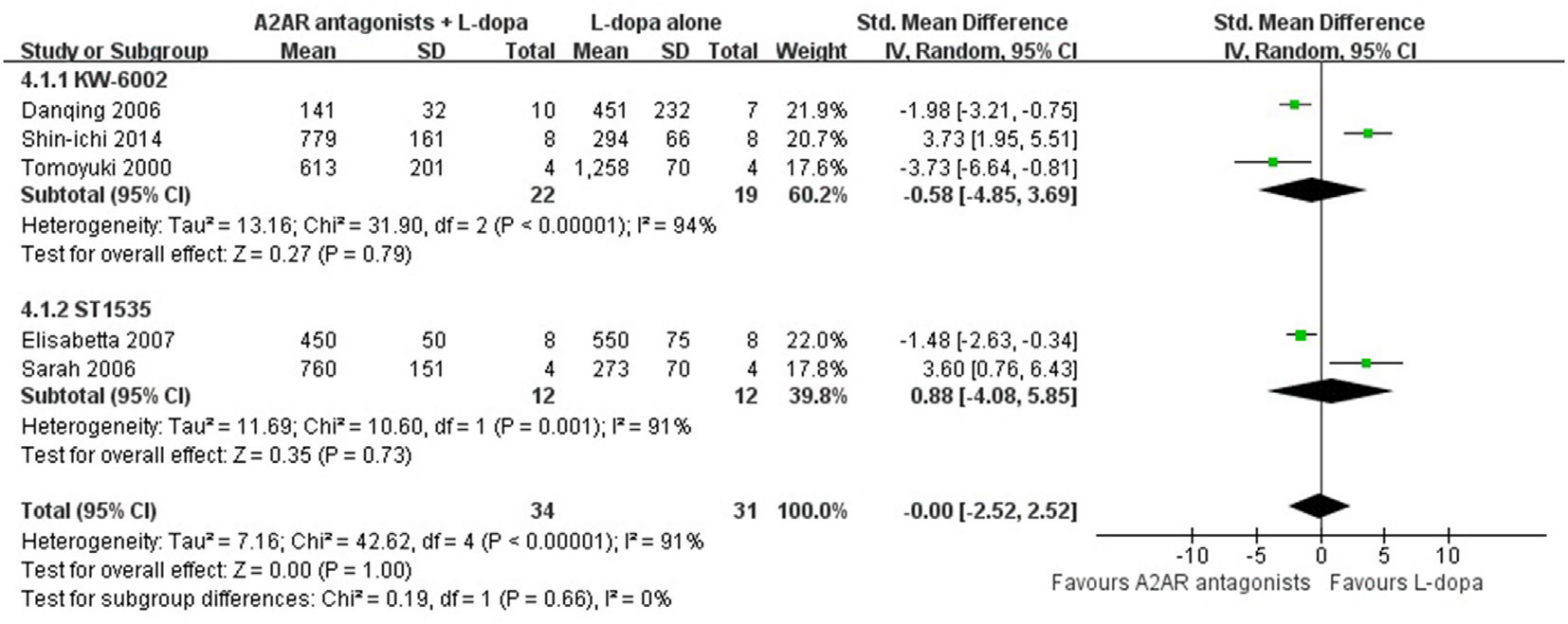
Subgroup analysis: the impact of different classes of A2A receptor antagonists (KW-6002 and ST1535) on the estimate of improvement in locomotor activity score outcome.

**Figure 4:**
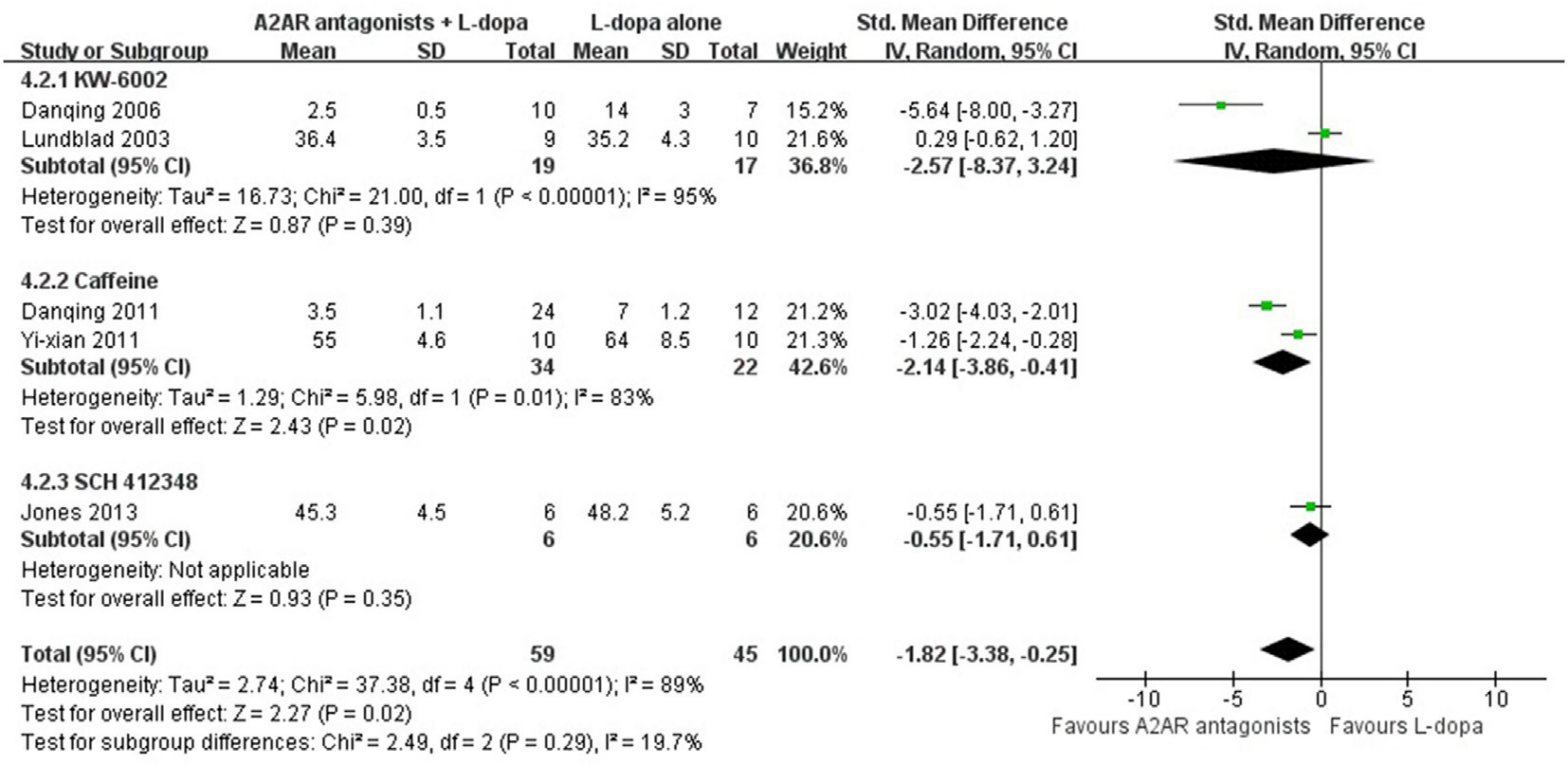
Subgroup analysis: the impact of different classes of A2A receptor antagonists (KW-6002, caffeine and SCH 412348) on the estimate of decrement in total abnormal involuntary movements (AIM) score outcome.

In the subgroup analysis focusing on locomotors activity, the efficacy of KW-6002 was similar with ST1535 (SMD −0.58, 95% CI: −4.85 to 3.69, *p* = 0.79; SMD 0.88, 95% CI: −4.08 to 5.85, *p* = 0.73, respectively, Figure 3). Moreover, in terms of total AIM score, efficacy was observed to be higher with the administration of Caffeine than KW-6002 or SCH 412348 (SMD −2.14, 95% CI: −3.86 to −0.41, *p* = 0.02 < 0.05; SMD −2.57, 95% CI: −8.37 to 3.24, *p* = 0.39; SMD −0.55, 95% CI: −1.71 to 0.61, *p* = 0.93, respectively, Figure 4).

## Discussion

### Summary of Main Results

This meta-analysis demonstrated that A2A receptor antagonists substantially reduced total AIM in animal models of LID. Meanwhile, we found A2A receptor antagonists plus l-dopa has similar anti-parkinsonism effects on the locomotors activity and motor disability scores when compared with l-dopa alone. Based on the subgroup analysis, the results showed that effect size of KW-6002 on the locomotors activity in animals was similar with ST1535. Moreover, we found that animals who received Caffeine showed remarkable improvement on the total AIM score compared with KW-6002 or SCH 412348. The improvement in LID behavior without an impact of motor function is highly significant as it differentiates A2A receptor antagonists from the known effects of dopaminergic therapies.

We conducted a preclinical animal meta-analysis for a better understanding and also to facilitate the conversion of experimental evidence to future clinical trials. With such aim, 6-OHDA and MPTP models were chosen as the pathophysiological model as they were closer to human LID. The analysis of A2A receptor antagonists in LID could be of great interest for future studies. However, these findings have to be interpreted with caution, because only a few different A2A receptor antagonists’ studies were tested in this paper. Nevertheless, the present results reinforced the neuroprotective role of A2A receptor antagonists in experimental LID models, but we did not sure whether it reliably informed human studies. Undoubtedly, further evidence was required to confirm this efficacy in human.

To date, alternative to modification of l-dopa therapy to avoid LID may resort to non-dopaminergic strategies of the indirect pathway of the basal ganglia. One possible strategy is to inactivate the A2A receptors (28). It has been displayed that the expression of adenosine A2A receptors was increased following l-dopa treatment and the appearance of AIM or LID as shown by biochemical, post-mortem, and imaging investigations, suggesting that inhibitation of A2A receptors is helpful for LID. Moreover, numerous preclinical studies have reported that A2A receptor antagonists might hinder the development of LID, which are consistent with this review (29). Moreover, clinical trials have showed that oral administration A2A antagonists increase functional on-time duration in PD patients suffering from wearing-off phenomenon (less effective than DBS or l-dopa continuous infusion), although they may increase dyskinesia in patients with advanced PD (14). Thus, critical aspects of the potential function of A2A antagonists on LID patients are yet to be evaluated.

### Interpretation of the Results

The interaction between adenosine A2A and D2 DA receptors has recently attracted much attention as a possible therapeutic target in LID (30). In addition, an increase in adenosine A2A receptor was observed in the striatum of dyskinetic primate’s models (31). A2A receptors were reported to adjust the activity of the indirect pathway and modulate acetylcholine and glutamate release in the striatum, where they form functional heteromeric complexes with D2 receptors (10). Namely, agonists of A2A receptors inhibit the binding of DA to D2 receptors and have been shown to inhibit D2-mediated neurotransmitter release. On the other hand, evidence shows that A2A receptors antagonists mimic the effects of D2 agonists (29). Interestingly, recent evidence demonstrates that A2A receptors co-localize postsynaptically not only with D2 receptors, but also with the cholinergic interneurons, the cannabinoid receptor and the glutamate receptor, suggesting the existence of important multifunctional interactions between A2A and these receptors (32). In summary, these data strongly indicate that A2A receptors can target a couple of cellular mechanisms involved in the underlying neurodegenerative process, and likely to play a functional role in the modulation of motor behavior in LID.

In our study, caffeine showed more remarkable improvement on the total AIM score when compared with other A2A antagonists. The underlying mechanism by which caffeine attenuates LID remains largely unclear. One previous study reported that chronic administration of l-dopa increased the striatal A2A expression significantly. Nevertheless, co-administration of l-dopa with caffeine not only attenuated the AIM induced by l-dopa, but also lower the level of striatal A2A expression (24). Moreover, Xiao et al. suggested that blocking both A1 and A2A receptors simultaneously, as occurred with caffeine use, might also confer a disease-modifying benefit of reduced risk of disabling LID, suggesting A1 may also play a pivotal role in the function of caffeine (25). On the other hand, SCH 412348 was given concurrently with l-dopa over the course of 19 days of treatment. This treatment paradigm did not reduce the severity of AIM score compared with treatment with l-dopa alone (*p* = 0.35). With regard to KW-6002, our data showed that KW-6002 could not prevent the development of AIM score even when the combined treatment is administered *de novo* (*p* = 0.39).

### Limitations

Several limitations of this meta-analysis should be considered. First, there is a chance of overestimation of the efficacy because our paper can only include available data which have been published in some forms, and hence negative studies that are less likely to be published will be missed. Therefore, the inclusion of unpublished studies and the use of trial registries become reasonable means to avoid publication bias (33). Second, a notable feature of the present review is the marked heterogeneity between studies due to the variation in study quality and experimental designs, implying that the overall estimate of efficacy should be interpreted with some caution. Meanwhile, this meta-analysis included a limited number of small studies (*n* = 9) and type-II errors due to chance cannot be entirely excluded as an alternative explanation for our main finding (34), making these findings less robust. Although there is no fixed minimum number of studies required for a meta-analysis, too small a number could lead to an unstable effect size. Therefore, further studies, particularly those of large sample, were warranted to support the drugs’ superiority to placebo. Third, our meta-analysis is based on observational research rather than experimental, and thus we are only able to obtain associations rather than causation. Moreover, no study in this meta-analysis using animals with co-morbidities, which is the typical situation in human PD and LID. Finally, as the studies only involved a few classes of A2A receptor antagonists, the majority being KW-6002 (*n* = 4), the results cannot be extrapolated to other A2A receptor antagonist’s classes.

### Implications for Further Studies

When included in systematic reviews, high-quality studies with lower variance will show larger effects, and improvement in the quality of reporting studies will also help to reduce bias. Therefore, well-designed and high-quality studies would be required to test the efficacy of A2A receptor antagonists on LID. In the present study, no studies investigated A2A receptor antagonists in LID models with concomitant conditions, such as hypertension, diabetes, dyslipidemia, or aged animals. This lack of information should certainly be addressed in future studies. Our meta-analysis suggested that the efficacy was maximal when Caffeine (*n* = 2, *p* = 0.02) was administered but not KW-6002 (*n* = 2, *p* = 0.39) or SCH 412348 (*n* = 1, *p* = 0.35) in terms of reduced the AIM score. However, the results generated from this subgroup analysis should be interpreted with caution due to the limited studies. We have no sufficient evidence to suggest initiating clinical trials based on these data. Consequently, further studies would be demanded to determine which kinds of A2A receptor antagonists were more effective than others. Moreover, there is currently little accordance on which neurobehavioral tests in rats would offer measures that are predictive of a benefit in clinical patients. In terms of PD, after a few years of l-dopa therapy, most patients will be accompanied with AIM (including movements with dystonic, choreiform, ballistic, or stereotypic features) that appear when plasma and brain levels of l-dopa are high, mimicking the peak-dose variant of human LID (35). It was long assumed that the responsiveness to l-dopa merely could be measured with contralateral rotation test but LID movements was unable to be assessed at all, until Cenci and collaborators first introduced the concept of AIM in 1998 (36). Although contralateral rotations have been used as a measure of LID, it has become increasingly recognized that this neurobehavioral not always correlates with the development of LID (37). Therefore, further studies should use AIM score as an indicator to reflect LID behavior.

## Conclusion

In summary, we have shown that adenosine A2A receptor antagonists are effective in the management of LID in animal models. Although some factors, such as study quality and total sample sizes, may undermine the validity of the positive findings, A2A receptor antagonists still probably have a potential neuroprotective role in LID models. The systematic review and meta-analysis here provides a framework for an evidence-based approach to the development of new treatments for LID and for the design of future preclinical and clinical studies.

## Author Contributions

M-MZ and LF conceived and participated in its design, searched databases, extracted and assessed studies, and helped to draft the manuscript. X-RZ, JC, and Z-RZ carried out the statistical analysis and interpretation of data. W-WW and C-LX participated in the conceptualization and design of the review, performed the selection of studies, data extraction and analysis, and drafted the review. All authors read and approved the final manuscript.

## Conflict of Interest Statement

The authors declare that the research was conducted in the absence of any commercial or financial relationships that could be construed as a potential conflict of interest.
